# *Euglena gracilis* and Its Aqueous Extract Constructed With Chitosan-Hyaluronic Acid Hydrogel Facilitate Cutaneous Wound Healing in Mice Without Inducing Excessive Inflammatory Response

**DOI:** 10.3389/fbioe.2021.713840

**Published:** 2021-12-10

**Authors:** Jin Li, Zezhou Zheng, Ming Du, Jinchun Chen, Hui Zhu, Zhangli Hu, Yanxia Zhu, Jiangxin Wang

**Affiliations:** ^1^ Shenzhen Key Laboratory of Marine Bioresource and Eco-Environmental Science, Shenzhen Engineering Laboratory for Marine Algal Biotechnology, Guangdong Provincial Key Laboratory for Plant Epigenetics, College of Life Sciences and Oceanography, Shenzhen University, Shenzhen, China; ^2^ Key Laboratory of Optoelectronic Devices and Systems of the Ministry of Education and Guangdong Province, College of Optoelectronic Engineering, Shenzhen University, Shenzhen, China; ^3^ College of Food Engineering and Biotechnology, Hanshan Normal University, Chaozhou, China; ^4^ Shenzhen Key Laboratory of Anti-Ageing and Regenerative Medicine, Health Science Center, Shenzhen University, Shenzhen, China

**Keywords:** wound healing, live Euglena gracilis cells, aqueous extract, microalgal therapeutics, chitosan-hyaluronic acid hydrogel

## Abstract

Naturally occurring compounds isolated from the microalga *Euglena gracilis*, such as polysaccharide paramylon, exhibit antimicrobial, anti-viral, antitumor, and anti-inflammatory activities. Whether live *E. gracilis* cells and its aqueous extract accelerate burn wound healing remains to be investigated. In this study, live *E. gracilis* cells and its aqueous extract were mixed with chitosan-hyaluronic acid hydrogel (CS/HA) to form cell + CS/HA and extract + CS/HA, which were then smeared onto the deeply burned skin of mice. The efficacy of these mixtures in accelerating wound healing was assessed through wound size reduction measurement, histological and immunofluorescence analyses, and serum pro-inflammatory cytokine level (INF-γ, IL-1β, and IL-6) determination. The live *E. gracilis* cells and its aqueous extract were found to facilitate wound healing by enhancing re-epithelization and reducing fibroplasia without stimulating excessive inflammatory response. In conclusion, live *E. gracilis* cells and its aqueous extract can be potentially used to treat cutaneous wounds.

## 1 Introduction

Acute skin wounds affect individuals physically and mentally. Annually, millions of people worldwide are affected by poor wound healing after surgery, trauma, acute illness, or chronic disease conditions (Eming et al., 2014). Although skin lesions heal rapidly and efficiently within 2 weeks, epidermal appendages (e.g., sebaceous glands and hair follicles) are not regenerated at the damaged site within this period, and a connective scar with a poorly reconstituted collagen matrix can be observed (Ehrlich and Krummel 1996; Almine et al., 2012). Therefore, the major goal of wound healing biology is to induce perfect reconstruction of damaged skin parts. Although various types of therapies have been tested to accelerate the wound healing process, optimal strategies are still being developed (Chen et al., 2018). Advances in microalgal therapeutics seem promising in promoting skin wound healing. A photoautotrophic cyanobacterium, *Synechococcus elongatus* PCC7942, accelerates wound healing by promoting angiogenesis (Yin et al., 2019). In several studies, *Euglena-*derived polysaccharide paramylon has shown promising results in tissue repair therapy (Sugiyama et al., 2010; Shibakami et al., 2013; Shibakami et al., 2015). Paramylon film accelerated skin wound healing in an animal model through its immunosuppressive effect (Yasuda et al., 2018).

Previous studies have reported that β-1,3-D-glucan modulated the Th1 and/or the Th2 cell response in experimental animals and human patients of allergic rhinitis and digestive cancers (Yoshino et al., 2000; Kirmaz et al., 2005; Ahmada et al., 2018, 2019). Leung and Bieber (2003) suggested a key role of the Th1-type cytokine interferon-γ (IFN-γ) in the chronicity of atopic dermatitis (AD) lesions of human. Oral administration of *Euglena-*derived polysaccharide paramylon inhibits the development of AD-like skin lesions in NC/Nga mice by suppressing both the T-helper (Th1) and Th 2 cell responses (Sugiyama et al., 2010). Serum levels of interleukin-4 (IL-4) and IFN-γ and IL-18 and IL-12 contents in the skin lesions were reduced. Moreover, sonicated and alkalized paramylon derived from *E. gracilis* upregulates pro-inflammatory factors (nitric oxide, tumor necrosis factor alpha, IL-6, and cyclooxygenase 2) in lymphomonocytes and has an immune-activating effect (Russo et al., 2016). Kankkunen et al. (2010) have demonstrated that paramylon as well as other large particulate β-1,3-D-glucans (curdlan, zymosan, glucan from baker’s yeast *Saccharomyces cerevisiae*) are sensed by sophisticated cooperating pathways through both membrane-bound and cytosolic pattern recognition receptors (PRRs), resulting in robust activation of IL-1β-mediated inflammatory response in human primary macrophages. In this study, immunosuppressive effect of live *Euglena gracilis* cells and its aqueous extract for facilitating skin wound healing was investigated in an animal model. The expression of three important pro-inflammatory mediators of antibody responses along different pathways such as IL-6, IFN-γ, and IL-1β was evaluated.

*E. gracilis* is a unicellular green microalga with flagellar motility (Buetow, 1968). The presence of nutritionally crucial chemicals such as fatty acids, docosahexaenoic acid, eicosapentaenoic acid, and vitamins in *E. gracilis* implies that this alga is a valuable therapeutic resource with potential for clinical application (Kottuparambil et al., 2019; Nakashima et al., 2021). The paramylon yield from *E. gracilis* is approximately 60%–70% of the dried cells, and paramylon exhibits anti-inflammatory, antimicrobial, antioxidant, anticancer, and neuroprotective properties, as well as immune activating effects (Sakagami et al., 1991; Foltínová et al., 1994; Sugiyama et al., 2009; Russo et al., 2016; Nakashima et al., 2017; Suzuki et al., 2018; Guo et al., 2019). On the other hand, methanol or ethanol extracts of *Euglena* species (e.g., *E. viridis*, *E. gracilis,* and *E. tuba*) possess antimicrobial, antiviral, and antitumor properties (Das et al., 2005; Panja et al., 2016; Ishiguro et al., 2020). As a cosmetic or dermopharmaceutic compound, the aqueous extract of *Euglena* activates cellular metabolism and reduces the signs of aging and cutaneous fatigue (US patent US8741357B2) (Lintner et al., 2014). The author reported a decrease in deformability and an increase in cutaneous vitality of skin after 14 days treatment with 3% *Euglena* extract. Nevertheless, *E. gracilis* components, other than paramylon, stimulate the growth of *Faecalibacterium* and improve digestive health (Nakashima et al., 2021). Hence, we investigated whether live *E. gracilis* cells and its aqueous extract exert beneficial effects on skin wound healing.

Chitosan-hyaluronic acid hydrogel (CS/HA) has good biocompatibility, and therefore, it can be used as a delivery device not only for mobilizing stem cells to the injection site, but also for sustainable release of bioactive molecules or growth factors (Zhu et al., 2017). CS/HA can provide a moist environment to the wound, thereby effectively preventing tissue dehydration and cell death, enhancing the migration of inflammatory cells and growth factors, facilitating air exchange and angiogenesis, serving as a barrier for microbes, removing excessive exudate, and accelerating wound healing (Luo et al., 2010; Rudyardjo and Wijayanto, 2017). In our preliminary study, paramylon + CS/HA, extracellular vesicle + CS/HA, cell + CS/HA, and extract + CS/HA were developed by incorporating sonicated and alkalized paramylon, extracellular vesicle, live *E. gracilis* cells, and its aqueous extract into CS/HA in a 1:1 volume ratio. Sonicated and alkalized paramylon, extracellular vesicle, live *E. gracilis* cells, and its aqueous extract were uniformly distributed in CS/HA. Cells survived in CS/HA after a 24-h incubation in an illuminating incubator without shaking at 37°C (data not shown). Keeping CS/HA as the control group, the aforementioned four groups were smeared onto the deeply burned skin of mice. Wound size reduction was calculated as follows: wound size reduction (%) = (A_0_ − At)/A_0_ × 100, where A_0_ is the initial wound area and At is the wound area at day 14 after wounding (Zhang et al., 2015). Results of wound size reduction measurement revealed that cell + CS/HA and extract + CS/HA facilitated wound healing more rapidly than paramylon + CS/HA, extracellular vesicle + CS/HA, and CS/HA. In the present study, we investigate the ability of live *E. gracilis* cells and its aqueous extract to accelerate wound healing based on the results of wound size reduction measurement, histological and immunofluorescence analyses, and serum pro-inflammatory cytokine level determination. To the best of our knowledge, this is the first study to investigate the direct effect of live *E. gracilis* cells and its aqueous extract on the wound healing process.

## 2 Materials and Methods

### 2.1 Isolation of Live *Euglena gracilis* Cells and Aqueous Extract

*E. gracilis* cells were grown in the EM medium under a light intensity of 100 μmol/m^2^/s in an illuminating incubator without shaking at 26°C until the cells reached the stationary phase (Afiukwa and Ogbonna, 2007; Wang et al., 2018). The medium contained 1.8 g/L NH_4_Cl, 0.6 g/L KH_2_PO_4_, 0.6 g/L MgSO_4_, 60 mg/L urea, 0.02 g/L CaCl_2_, 0.48 mg/L Na_2_EDTA, 2 mg/L Fe_2_(SO4)_3_, 60 μl HCl, 0.01 mg/L Vb_1_, 0.0005 mg/L Vb_12_, 20 mg/L CuSO_4_·5H_2_O, 0.4 g/L ZnSO_4_·7H_2_O, 1.3 g/L Co(NH_3_)·H_2_O, and 1.6 g/L MnCl_2_·4H_2_O.

The *E. gracilis* culture medium was incubated for 1 week, and then, 1 L of this medium was centrifuged at 1,000 × *g* for 4 min and collected. Cell precipitates were rinsed three times with distilled water and resuspended in 1× phosphate buffered saline (PBS) at 37°C. After cultivating the cells for 1 week, the aqueous extract was isolated from 1 L *E. gracilis* culture medium. Briefly, the culture was centrifuged at 7,000 × *g* for 3 min, and the cell pellets were rinsed three times with distilled water and then ultracentrifuged at 10,000 × *g* for 5 min three times. An ultrasonic cell pulverizer (model: JY99-IIDN, Ningbo Scientz Biotechnology Co., Ltd.) was used as an emulsified dispersion device (70% power). The precipitated cells with 20 volumes of 1× PBS were ultrasonically treated for 8 min. The device adopts a working time of 48 s and intermittent 12 s, which can effectively prevent temperature increase and improve emulsion efficiency. The cellular debris was removed using a 0.22-μm Millipore filter.

### 2.2 Preparation of Chitosan-Hyaluronic Acid Hydrogel

Chitosan-hyaluronic acid hydrogel was prepared according to the method of Zhu et al. (2017). Briefly, a 2% chitosan (CS, deacetylation 90%, Sigma) stock was prepared in 0.1 M hydrochloric acid, and a 10% β-glycerophosphate (GP, Sigma) stock and a 1% sodium hyaluronate (HA, 350 kDa, Huaxi Fureida) stock were prepared using distilled water. The 2% CS, 10% GP, and 1% HA solutions were subsequently mixed and maintained in a 37°C water bath before use. The hydrogel prepared with the proportions of CS:GP:HA = 5:3:2 was chosen as the optimal gel to promote cell proliferation and differentiation because it displayed good mechanical properties.

### 2.3 Mouse Skin Wound Model and Treatments

The Animal Research Committee of the Health Science Center of Shenzhen University approved all experimental procedures. Successful skin wound healing involves a series of events with complex cell signaling cascades that coordinate several fundamental biological processes (Martin 1997; Gurtner et al., 2008). Re-epithelialization, granulation tissue formation with collagen deposition, and successive influx of different subsets of immune cells match the classical wound healing timeline in BALB/c mice (Braiman-Wiksman et al., 2007). Briefly, 1–3 days post-wounding stage included blood-clot formation (primary clot), activation of epidermal edges, and early inflammatory response (characterized by abundance of neutrophils at the wound gap). Four to seven days post-wounding stage was marked by scab formation. Histological analysis reveals migration of the epidermal edges, selective proliferation of the early granulation tissue, and inflammatory response (lymphocytes and macrophages present in abundance). Scar detachment is observed at 8–12 days post-wounding stage. Histological results exhibit the formation of new epidermis and initiation of dermal closure. This stage is accompanied by attenuation of the inflammatory response. However, epidermal closure progresses considerably more slowly. For example, at 12 days following wounding, 40% of the wounds exhibit dermal closure (Braiman-Wiksman et al., 2007). Therefore, in the present study, we select 14 days post-wounding as the third stage. Critical events of the stepwise experimental wound healing process at 1, 7, and 14 days were probed. Wound healing did not diverge from the histomorphological features of this paradigm.

In total, 22 female 8-week-old BALB/c mice (weight: 16–20 g) were anesthetized through intraperitoneal injection of 4% chloral hydrate (1 ml/100 g). After shaving the mice, two dorsal wounds were symmetrically clipped out using a copper bar (diameter: 1 cm) punch, which was heated using a water bath at 95°C.

The mice were randomly assigned to three groups: cell + CS/HA (5 × 10^6^ live *E. gracilis* cells mixed with CS/HA in 1:1 volume ratio), extract + CS/HA (0.05 g/ml aqueous extract from *E. gracilis* mixed with CS/HA in 1:1 volume ratio), and CS/HA (control group). A total of 42 wound sites (14 wound sites/group) were analyzed.

### 2.4 Histological Study

The mice were sacrificed at day 14 after wounding. For histological analyses, the skin excised from 21 dorsal wound sites (7 sites/group) was fixed in a 4% paraformaldehyde fix (PFA) solution, dehydrated with a graded alcohol series, embedded in paraffin, sectioned (section thickness: 4 μm) perpendicularly to the wound surface, and stained with hematoxylin and eosin (H&E). Masson’s trichrome staining was used to determine the degree of collagen maturity.

### 2.5 Immunofluorescence Study

To identify vascular structures, immunofluorescence histochemistry was performed for an endothelial cell marker, CD31. For immunofluorescence staining, skin excised from the dorsal wound sites was fixed in 4% PFA (cat no. BL539A, Biosharp), dehydrated in a 30% sucrose solution, embedded in optimal cutting temperature compound (OCT), and sectioned (section thickness: 4 μm) perpendicularly to the wound surface. Tissue sections were blocked in 5% BSA for 30 min at room temperature and incubated with rabbit CD31 monoclonal antibody (1:200, ab28364, Abcam) overnight at 4°C (Zhu et al., 2021). Images were acquired using an Olympus IX81 microscope. The newly formed and mature vessels were indicated by CD31 positive staining. These newly formed vessels were counted in five random fields per section between wound edges by using ImageJ (v. 1.52) (Schneider et al., 2012).

### 2.6 Enzyme-Linked Immunosorbent Assay for IFN-γ, IL-1β, and IL-6

The effect of live *E. gracilis* cells and its aqueous extract was examined by investigating serum pro-inflammatory cytokine levels through enzyme-linked immunosorbent assay (ELISA). In total, 15 blood samples (5 samples/group) were obtained from the inferior vena cava of the mice under anesthesia on day 14. IL-1β (cat no. 88-7013) and IL-6 (cat no. 88-7064) levels were measured using the ELISA Development Kit, according to the manufacturer’s protocols (Multi Sciences [Lianke] Biotech Co., Ltd., Hangzhou, China). Each sample was measured in duplicate, and cytokine concentration was calculated on the basis of standard curves provided with the kits. The results are expressed in pg/ml. IFN-γ (cat no. JM-02465M1) was measured using the ELISA Development Kit, according to the manufacturer’s protocols (Jingmei Biotech Co., Ltd., Yancheng, China). Because the IFN-γ level is excessively high, each sample was diluted 5-fold, and the results were multiplied by the dilution factor (5-fold). As serum available was insufficient, IL-6 concentration was detected for one sample in each group.

### 2.7 Statistical Analysis

Statistical significance was evaluated using GraphPad Prism (v7, GraphPad Software, Inc., La Jolla, CA, USA), and statistical analysis was performed using analysis of variance (ANOVA) followed by the Tukey’s test for post-hoc analysis. *p* value less than 0.01 (*p* < 0.01) was considered extremely significantly different, *p* < 0.05 was statistically significant.

## 3 Results

### 3.1 Wound Size Reduction Was Significantly Greater in Cell + CS/HA and Extract + CS/HA

**FIGURE 1:**
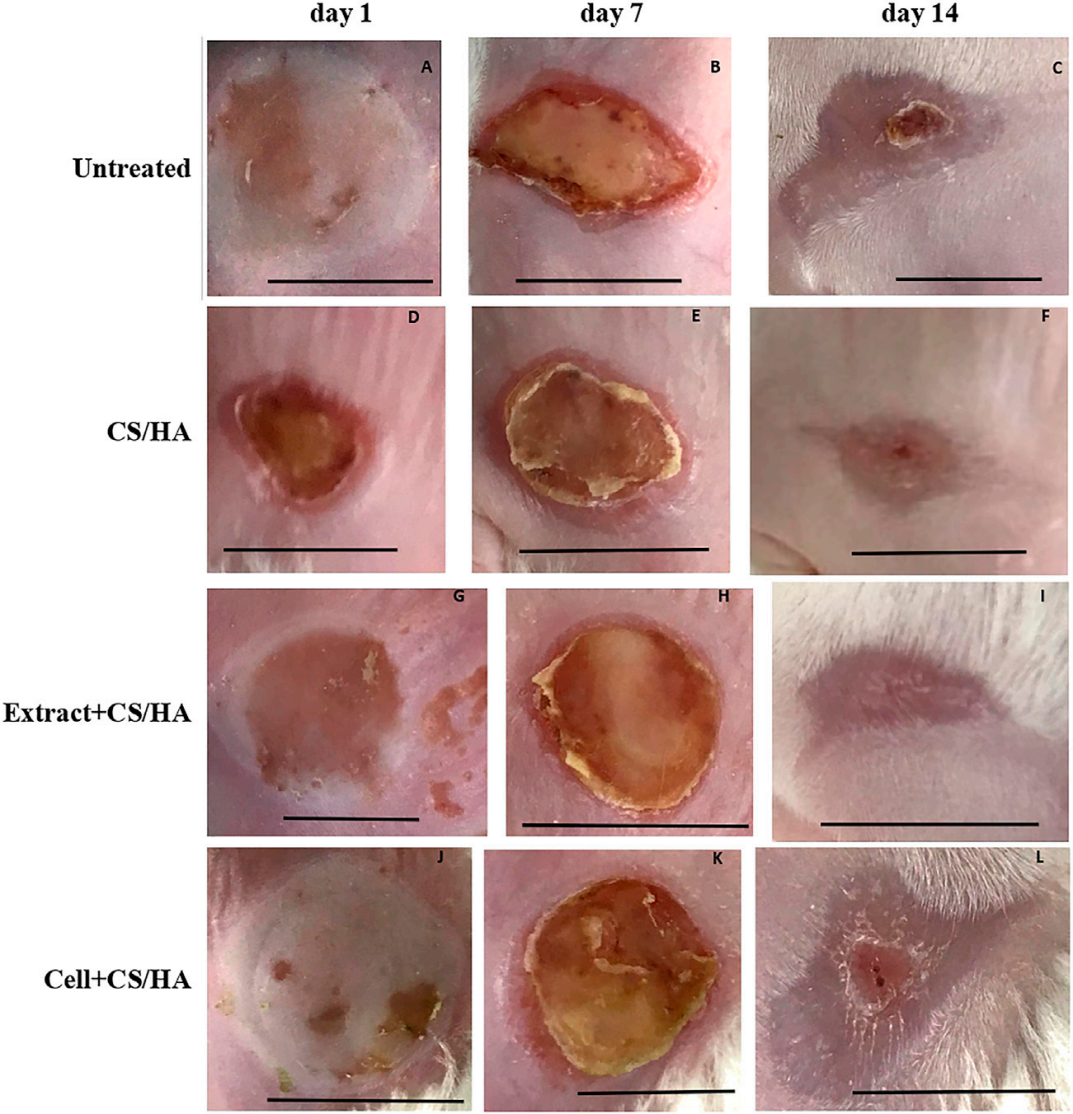
Macroscopic appearance of wounds treated with CS/HA, extract + CS/HA, and cell + CS/HA at 1, 7, and 14 days in the mice. **(A–C)** Untreated (*n* = 1). **(D–F)** CS/HA (*n* = 7). **(H–J)** Extract + CS/HA (*n* = 7). **(K, L)** Cell + CS/HA (*n* = 7). Scale bar = 1 cm.

**FIGURE 2:**
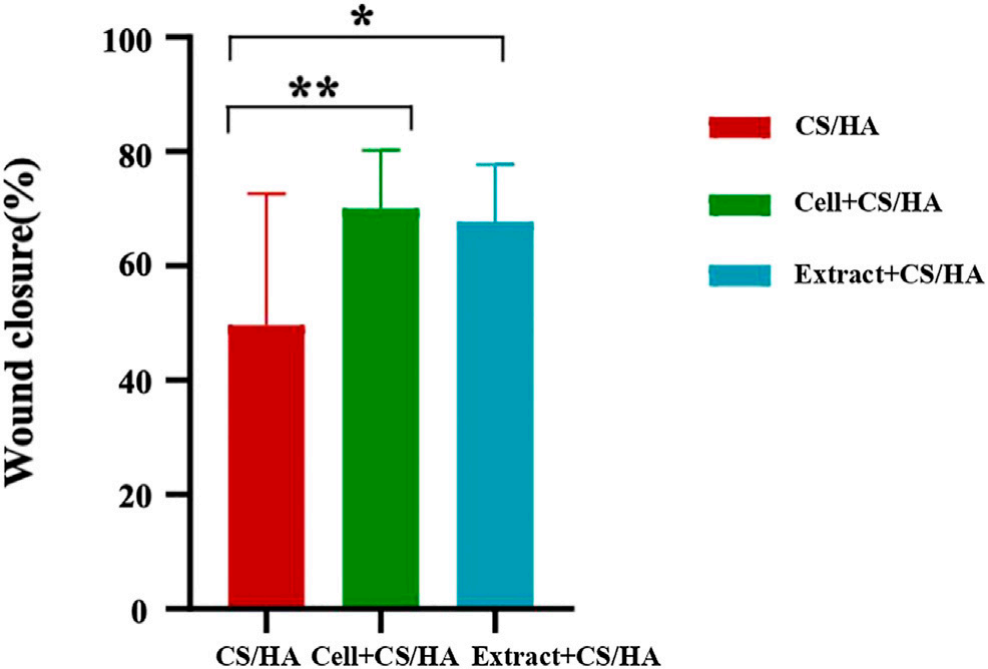
Comparison of wound size reduction among CS/HA, extract + CS/HA, and cell + CS/HA treatments at 14 days post-wounding. Wound size reduction was significantly greater in extract + CS/HA and cell + CS/HA. Significant difference compared to CS/HA. *p < 0.05, **p < 0.01. *n* = 14 per group.

Gross observations revealed an increase in size reduction of wounds treated with cell + CS/HA and extract + CS/HA compared with those treated with CS/HA at 14 days post-wounding (Figure 1). Wounds treated with cell + CS/HA and extract + CS/HA exhibited a significantly greater reduction in size than those treated with CS/HA (approximately 70 and 67%, respectively, vs. 49%; Figure 2).

### 3.2 Enhanced Re-Epithelialization and Reduced Fibroplasia in Cell + CS/HA and Extract + CS/HA

**FIGURE 3:**
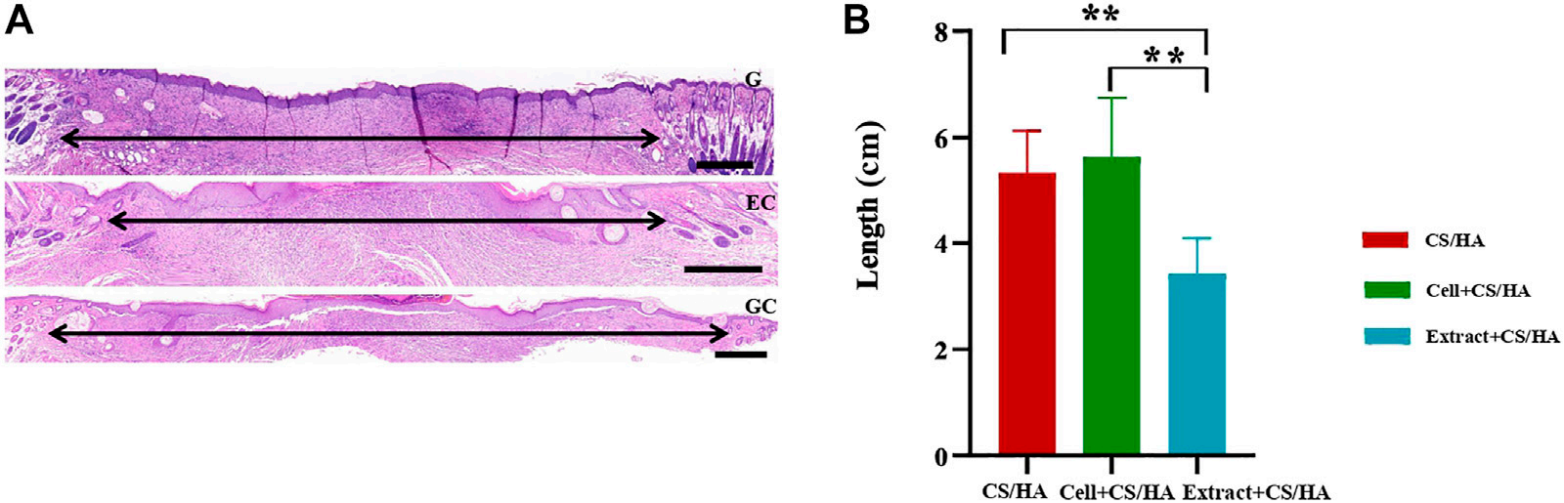
Representative H&E-stained images of wound sections and comparison of scar length among wounds treated with CS/HA, cell + CS/HA, and extract + CS/HA at 14 days post-wounding. **(A)** Representative images of H&E-stained wound sections. Black double-headed arrows indicate the edges of scars. G: CS/HA; EC: extract + CS/HA; GC: cell + CS/HA. Scale bar = 500 μm. **(B)** Comparison of scar length after H&E staining. Wounds treated with extract + CS/HA had significantly narrower scar length than those treated with cell + CS/HA and CS/HA. Significant difference compared to CS/HA. *p < 0.05, **p < 0.01. *n* = 7 per group.

Reduced scar length and increased collagen maturity were used to assess the wound healing degree (Zhang et al., 2015). Both cell + CS/HA and extract + CS/HA enhanced re-epithelialization compared with CS/HA at 14 days post-wounding (Figure 3). H&E staining showed that wounds treated with extract + CS/HA had significantly narrower scar length than those treated with cell + CS/HA and CS/HA (Figure 3B). Additionally, wounds treated with cell + CS/HA showed a larger scar length than those treated with CS/HA; however, the difference was nonsignificant (Figure 3B).

**FIGURE 4:**
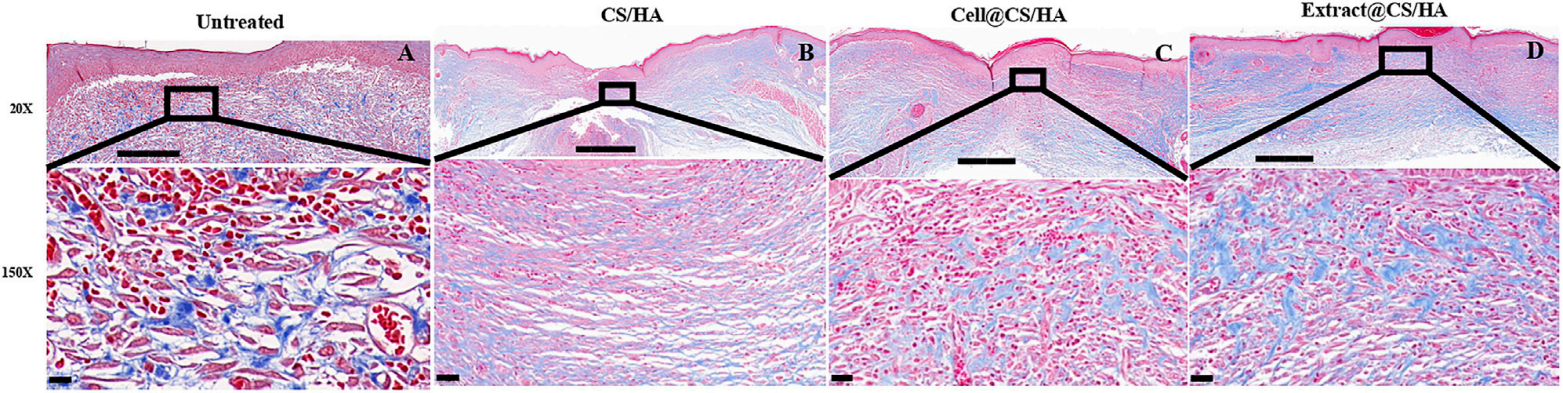
The representative Masson’s trichrome images of untreated group (*n* = 1) **(A)**, CS/HA (*n* = 7) **(B)**, extract + CS/HA (*n* = 7) **(C)**, and cell + CS/HA (*n* = 7) **(D)**. Wound tissue was stained with Masson’s trichrome to access collagen content and organization at 14 days post-wounding. Scale bar: 200 μm **(top)** or 10 μm **(bottom)**.

Dermal fibroblasts could secrete collagen and promote collagen deposition in the healing process (Li et al., 2016). In the present study, each group exhibited different amounts of collagen at 14 days post-wounding. The collagen fiber bundles were evident in the untreated group (Figure 4A). The application of CS/HA caused thin, undulated bundles of collagen in the scar tissue (Figure 4B). There was a lesser degree of fibroplasia in the extract + CS/HA and cell + CS/HA groups in comparison to the untreated group (Figures 4C,D).

**FIGURE 5:**
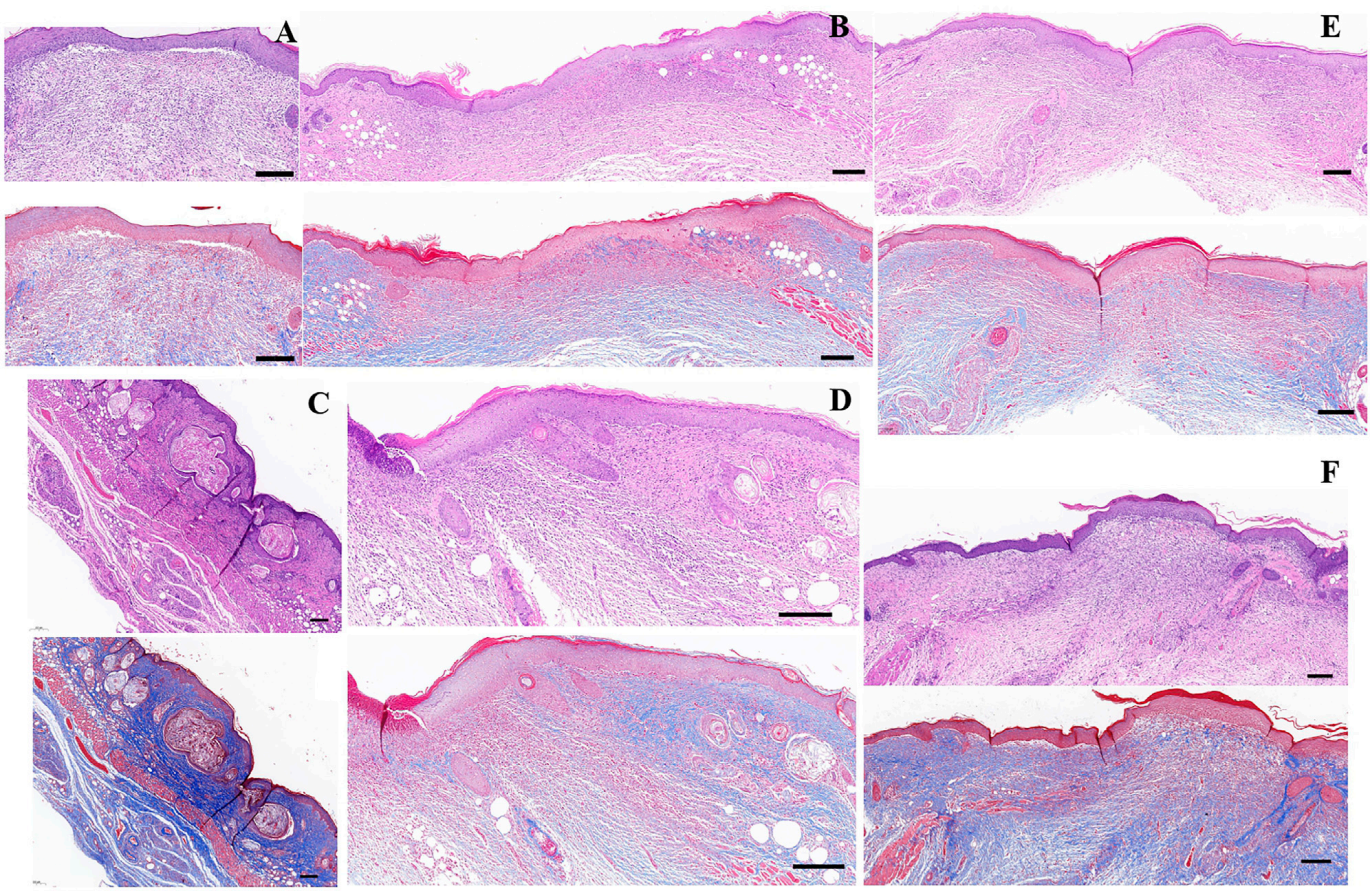
H&E- and Masson’s trichrome-stained images of wounds treated with CS/HA, extract + CS/HA, and cell + CS/HA at 14 days post-wounding. **(A)** Untreated tissue. **(B)** CS/HA. **(C,D)** Extract + CS/HA. **(E,F)** Cell + CS/HA. Scale bar = 200 μm.

Masson’s trichrome staining showed that wounds treated with cell + CS/HA (Figures 5A,B) and extract + CS/HA (Figures 5C,D) had more newly formed vessels than those treated with CS/HA, which only exhibited epidermis and dermis formation (Figure 5C). Inflammatory cells were not present in the dermis among wound sites treated with cell + CS/HA, extract + CS/HA, and CS/HA (Figure 5). Vascularization of newly formed tissues is essential for the wound healing process (Zhang et al., 2015). Newly formed vessels at wound sites were characterized by CD31 staining, from which average vessel densities were quantified. Herein, based on the enumeration of the newly formed vessels, no significant difference was noted in vessel density (Supplementary Figures S1A–D).

### 3.3 No Notable Changes in Cytokines (IFN-γ and IL-1β) in the Mice Treated With Cell + CS/HA and Extract + CS/HA

**FIGURE 6:**
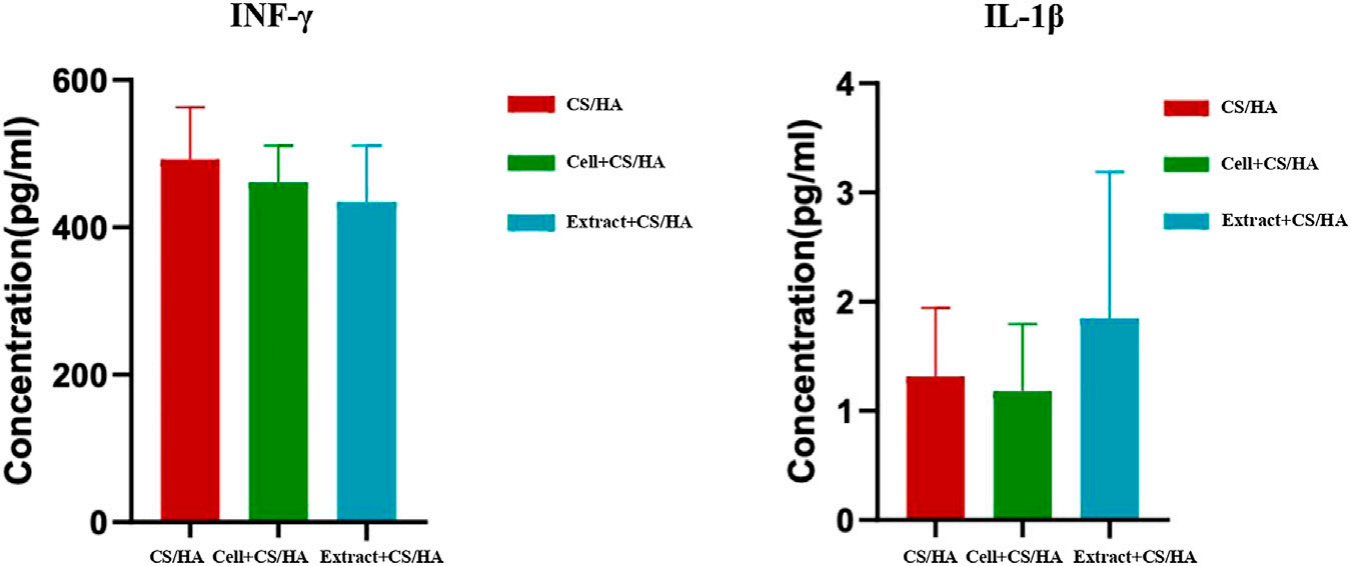
Comparison of INF-γ and IL-1β levels among wounds treated with CS/HA, extract + CS/HA, and cell + CS/HA at 14 days post-wounding. No notable changes in INF-γ and IL-1β levels in the mice treated with cell + CS/HA and extract + CS/HA.

The effect of cell + CS/HA, extract + CS/HA, and CS/HA applications on serum pro-inflammatory cytokines in the mice was investigated through ELISA. Cell + CS/HA and extract + CS/HA did not induce notable changes in IFN-γ and IL-1β (Figure 6) levels. Because serum available was insufficient, IL-6 concentration was detected for one sample per group (cell + CS/HA: 2.3 pg/ml; extract + CS/HA: 2.588 pg/ml; CS/HA: 0.959 pg/ml).

## 4 Discussion

Our study showed that live *E. gracilis* cells and its aqueous extract accelerated wound healing in the mice without inducing excessive inflammatory response. The cell + CS/HA and extract + CS/HA treatments led to greater wound size reduction and re-epithelialization, lesser degree of fibroplasia, and enhancement of new blood vessel formation in the wound bed. Both cell + CS/HA and extract + CS/HA treatments did not induce excessive inflammatory response, as revealed by no notable changes in IFN-γ and IL-1β concentrations. Moreover, inflammatory cells were not present in the dermis. Our findings that live *E. gracilis* cells and its aqueous extract facilitate wound healing without excessive immune responses are inconsistent with those obtained with paramylon of *E. gracilis*, which accelerates wound healing through its anti-inflammatory effect (Sugiyama et al., 2009; Sugiyama et al., 2010; Yasuda et al., 2018).

Live cells of cyanobacterium, *Synechococcus elongatus* PCC 7942, accelerate cutaneous wound healing by promoting angiogenesis (Yin et al., 2019). These cells exhibit the potential for wound healing primarily through the delivery of functional extracellular vesicles and not through their photosynthetic activity. Moreover, the promotion of IL-6 expression may be a mechanism for the pro-angiogenic effect and wound healing (Yin et al., 2019). As the existence of extracellular vesicles was confirmed in other microalgae (Picciotto et al., 2021), it is reasonable to propose similar mechanisms for live *Euglena* cells for skin wound healing. Additional elaborately designed experiments would provide strong evidence to support this case.

Although no study has reported the application of live *Euglena* cells, its aqueous extract in cosmetic compositions acts as an energy supplement for skin and hair follicle cells (US patent US8741357B2) (Lintner et al., 2014). The aqueous extract enables the reconstitution of the intracellular ATP pool and *de novo* synthesis of inositol 1,4,5-trisphosphate (IP3, a key molecule in the cell energy cascade), and it also triggers the release of intracellular calcium from its storage site (showing the switch of a cell from the resting state to the activated state) (US patent US8741357B2) (Lintner et al., 2014). The author also proposed that the aqueous extract of *Euglena* increases the energy supply to cells and/or tissues through the supply of phosphoinositides, which contribute to cell metabolism activation. It also stimulates the production of some biomolecules, such as ATP and calcium, in skin cells (US patent US8741357B2) (Lintner et al., 2014). Furthermore, the extract of *Euglena* species possesses a range of antimicrobial activities and/or the ability to increase cutaneous vitality (Das et al., 2005; Lintner et al., 2014). A high abundance of lipid-like, protein-like, lignin/polyphenol-like, carbohydrate-like, tannin-like compounds, and unsaturated hydrocarbon was found in the aqueous extract of light-grown *E. gracilis* cells (Lewis and Guéguen, 2020). In our laboratory, gas chromatography-mass spectrometry/mass spectrometry (GC−MS/MS) of the *Euglena* extract revealed the presence of hundreds of 2-3-amino-acid short peptides (data not shown). Short peptides have also been used as active elements for the detection of their own receptors (Pavan and Berti, 2012). For example, antimicrobial peptides (AMPs) and cell-penetrating peptides (CPPs) are used for sensing bacterial cells, antigenic peptide sequences for antibody monitoring, and peptide substrates for enzyme detection. Detailed investigations of the composition of the *Euglena* extract and their functions are being preceded to verify this hypothesis.

Signal transducer and activator of transcription 3 (STAT3) is the key mediator of both chronic inflammation and joint destruction in rheumatoid arthritis (RA). The major pro-inflammatory cytokines in RA, TNF-α, IL-6, and IL-1 induced STAT3 activation either directly or indirectly and stimulated expression of IL-6 family cytokines and receptor activator of nuclear factor kappa B ligand (RANKL) in an autocrine/paracrine manner *in vivo* and *in vitro*. Pharmacological inhibition of STAT3 also inhibited expression of RANKL in osteoblastic cells induced by IL-1β, TNF-α, and IL-6 *in vitro* as well as in the joints of a collagen-induced arthritis (CIA) model *in vivo* (Mori et al., 2011). In this study, no notable changes in IFN-γ and IL-1β concentrations were revealed by both cell + CS/HA and extract + CS/HA treatments. In addition, although IL-6 concentration was detected for one sample in each group, concentration values were similar (cell + CS/HA: 2.3 pg/ml; extract + CS/HA: 2.588 pg/ml). Thus, our data provide new insight into wound healing pathogenesis and provide evidence that inflammatory cytokines did not trigger a cytokine amplification loop.

A limitation of our study is that we did not identify the molecular mechanism underlying the pro-angiogenic and pro-wound healing effects of live *E. gracilis* cells and its aqueous extract. The molecules that mediate the different effects of live *E. gracilis* cells and its aqueous extract on the proliferation and migration of endothelial cells, keratinocytes, and fibroblasts also remain unknown. Thus, live *E. gracilis* cells and its aqueous extract may also be selectively enriched in some functional molecules that mediate their regulatory effects, although this approach requires further exploration.

Our findings suggest that live *E. gracilis* cells and its aqueous extract can facilitate wound healing without stimulating excessive inflammatory response during the healing process. This study is the first to provide evidence for the potential of live *E. gracilis* cells and its aqueous extract in treating cutaneous wounds. The successful wound healing in mice suggests the potential use of *E. gracilis* in humans for wound care.

## Data Availability

The datasets presented in this study can be found in online repositories. The names of the repository/repositories and accession number(s) can be found below: PRJNA734000.
