# Supplementary figures and images for: Euglena gracilis and Its Aqueous Extract Constructed With Chitosan-Hyaluronic Acid Hydrogel Facilitate Cutaneous Wound Healing in Mice Without Inducing Excessive Inflammatory Response
Source: Front Bioeng Biotechnol. 2021 Dec 10;9:713840. doi: 10.3389/fbioe.2021.713840 (PMC8703163; doi:10.3389/fbioe.2021.713840)

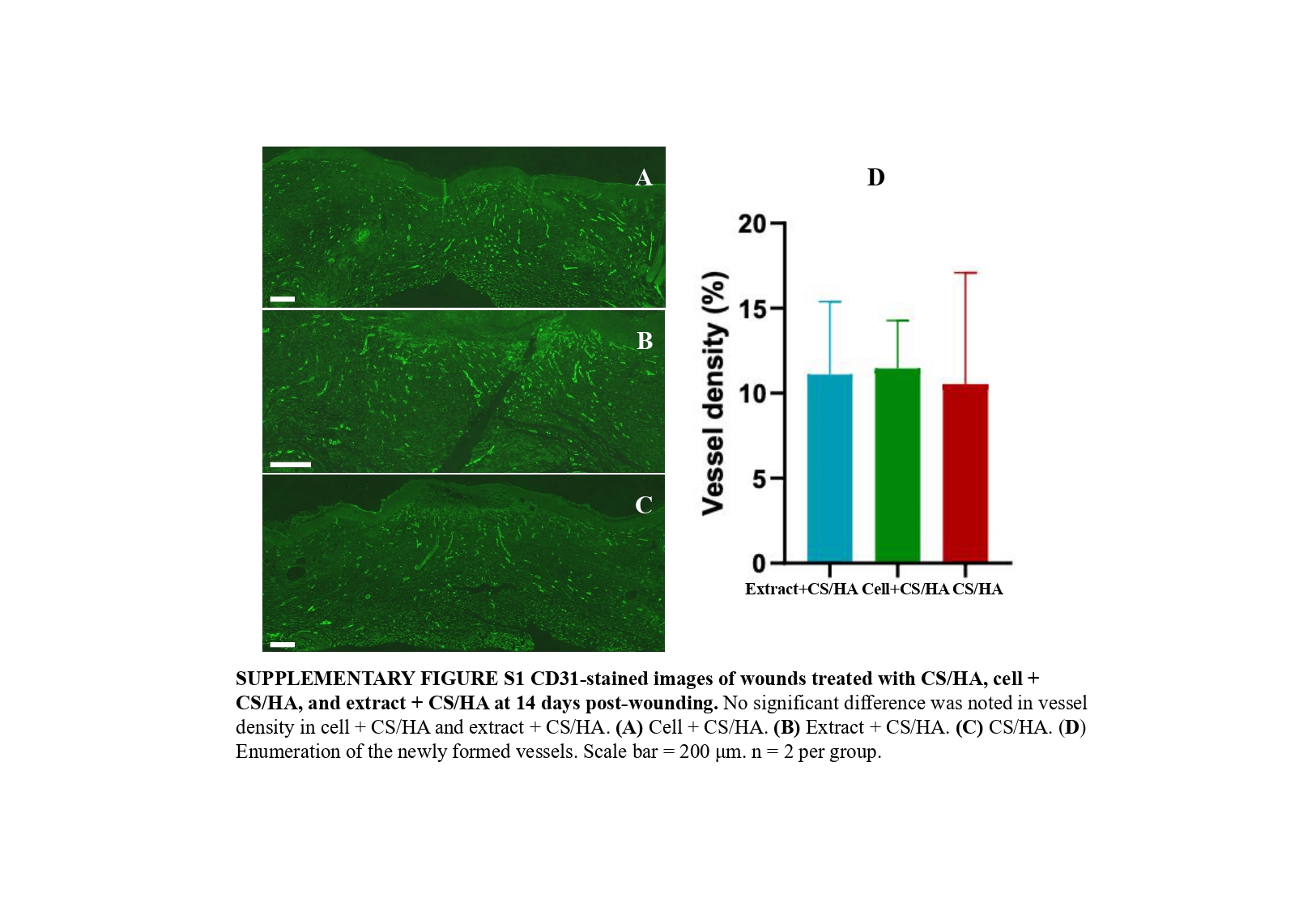

Supplement: Supplementary file 1 [file Image1.JPEG]
